# 2358: Association of medical and psychosocial risk factors with engagement in prenatal home visiting

**DOI:** 10.1017/cts.2017.104

**Published:** 2018-05-10

**Authors:** Kelly M. Bower, Deborah Gross, Margaret Ensminger, Jana Goins, Phyllis Sharps

## Abstract

OBJECTIVES/SPECIFIC AIMS: The purpose of this study is to understand factors that are associated with identifying which eligible pregnant women in Baltimore City accept a referral for HV services. Taking into account demographic and obstetrical variables, we will examine the extent to which 13 medical and 14 psychosocial risk factors differentiate pregnant women who (1) accepted a HV referral, (2) could not be located, or (3) refused a HV referral. METHODS/STUDY POPULATION: In this observational study, we will use secondary data on 8172 pregnant women collected by Health Care Access Maryland (HCAM) between 2014 and 2016. HCAM is the single point of entry for all pregnant women in Baltimore City into HV. HV eligibility includes being a pregnant woman, residing in Baltimore City, being uninsured or receiving Medicaid, and being identified by a prenatal care provider who completed an assessment profile of the woman’s medical and psychosocial risk (prenatal risk assessment). The outcome variable, HV engagement status (ie, accepted referral, could not be located, refused referral), will be based on HCAM discharge codes. Medical risk factors include BMI, hypertension, anemia, asthma, sickle cell, diabetes, vaginal bleeding, genetic risk, sexually transmitted disease, last dental visit >1 year ago, and taking prescription medications. Psychosocial risk factors include current pregnancy unintended; <1 year since last delivery; late entry to prenatal care (>20 wk gestation); mental, physical, or developmental disability; history of abuse or violence within past 6 months; tobacco use; alcohol use; illegal substance use within the past 6 months; resides in home built before 1978; homelessness; lack of social/emotional support; exposure to long-term stress; lack of transportation; and history of depression or mental illness. All risk factor variables are categorical (yes/no). Control variables will include demographics (eg, age, race, ethnicity, marital status, educational level) and OB history (eg, history of preterm labor, history of fetal or infant death). We will conduct descriptive statistics to characterize the sample and look for interrelatedness among the risk factors. Where there is a high level of inter-relatedness we will consider combining or omitting variables to reduce redundancy. We will use multinomial regression to examine which medical and psychological factors are associated with referral category. RESULTS/ANTICIPATED RESULTS: We hypothesize that (a) women with more medical risk factors will be more likely to accept a referral for HV services, (b) women with more psychosocial risk factors will be more likely to refuse HV or not be located, and (c) certain risk factors, such as depression/mental illness, history of abuse/violence, illegal substance use, homelessness, and exposure to long-term stress will be the strongest predictors of not accepting HV referral and/or not being located. DISCUSSION/SIGNIFICANCE OF IMPACT: The translation of effective randomized control trials (RCTs) to successful implementation in community-based programs can be challenging. Community-based programs serving low-income communities typically lack the same resources available to recruit and retain participants in RCTs. And, exclusion criteria applied in RCTs are often not applied in real world implementation which can open program to participants with more complex social and medical characteristics. Findings from this study will inform the translation of evidence-based HV programs into real world settings through an enhanced understanding of the characteristics of women who are not engaged by HV programs. This will inform development of improved outreach methods that may more effectively engage at-risk women for prenatal HV services.

